# L-Theanine: A Unique Functional Amino Acid in Tea (*Camellia sinensis* L.) With Multiple Health Benefits and Food Applications

**DOI:** 10.3389/fnut.2022.853846

**Published:** 2022-04-04

**Authors:** Ming-Yue Li, Hong-Yan Liu, Ding-Tao Wu, Ahmad Kenaan, Fang Geng, Hua-Bin Li, Anil Gunaratne, Hang Li, Ren-You Gan

**Affiliations:** ^1^Key Laboratory of Coarse Cereal Processing, Ministry of Agriculture and Rural Affairs, Sichuan Engineering & Technology Research Center of Coarse Cereal Industrialization, School of Food and Biological Engineering, Chengdu University, Chengdu, China; ^2^Research Center for Plants and Human Health, Chengdu National Agricultural Science and Technology Center, Institute of Urban Agriculture, Chinese Academy of Agricultural Sciences, Chengdu, China; ^3^National Graphene Institute, The University of Manchester, Manchester, United Kingdom; ^4^Guangdong Provincial Key Laboratory of Food, Nutrition, and Health, Department of Nutrition, School of Public Health, Sun Yat-sen University, Guangzhou, China; ^5^Faculty of Agricultural Sciences, Sabaragamuwa University of Sri Lanka, Belihuloya, Sri Lanka

**Keywords:** L-theanine, tea, health benefits, mechanisms of action, food applications

## Abstract

Tea (*Camellia sinensis L.*) is a very popular health drink and has attracted increasing attention in recent years due to its various bioactive substances. Among them, L-theanine, a unique free amino acid, is one of the most important substances in tea and endows tea with a special flavor. Moreover, L-theanine is also a bioactive compound with plenty of health benefits, including antioxidant, anti-inflammatory, neuroprotective, anticancer, metabolic regulatory, cardiovascular protective, liver and kidney protective, immune regulatory, and anti-obesity effects. Due to the unique characteristics and beneficial functions, L-theanine has potential applications in the development of functional foods. This review summarized the influencing factors of L-theanine content in teas, the main health benefits and related molecular mechanisms of L-theanine, and its applications in food, understanding of which can provide updated information for the further research of L-theanine.

## Introduction

Tea (*Camellia sinensis* L) is originated from China and is one of three major popular beverages in the world (1). Fresh tea leaves need to go through various processing procedures to be made into tea products prior to consumption. The processing operations, such as fermentation and baking, can change the color, aroma, taste, and chemical composition of tea (2, 3). Based on the degree of fermentation, tea can be divided into six categories, including green, yellow, white, oolong, black, and dark teas (4). Tea is rich in diverse chemical components, endowing tea with multiple beneficial functions (5, 6).

L-theanine, a non-protein water-soluble amino acid, is characteristically found in tea plants (7, 8). It is a unique taste component with caramel flavor, which can alleviate the bitterness of caffeinel (9). As a unique secondary metabolite in tea, L-theanine is the main source of tea flavor (10). L-theanine was proved to contribute to the generation of tea volatiles, which may be the main source of the crispy-rice-like smell and the chestnut-like balminess (11, 12). It can be used as one of the significant indexes to estimate the freshness of tea (13). In addition, it has many health benefits, such as antioxidant, anti-inflammatory, neuroprotective, anti-cancer, anti-anxiety, metabolic regulatory, cardiovascular protective, liver and kidney protective, and immune regulatory effects (14–16). Due to its flavor and diverse health benefits, L-theanine has wide applications, such as being used as a beverage ingredient or dietary supplement (17).

In our current review article, high-quality literature published in recent 5 years was collected from the Web of Science Core Collection and PubMed databases. The influence factors of L-theanine content in tea and its effect on tea fragrance were first introduced, and its health benefits were then summarized, with intensive discussion about the molecular mechanism of actions, and finally, its practical applications in foods were briefly introduced. We hope that this review paper can provide an updated understanding of L-theanine and support its wide applications in the development of L-theanine-based functional foods.

## Influencing Factors of L-Theanine Content in Tea

**FIGURE 1:**
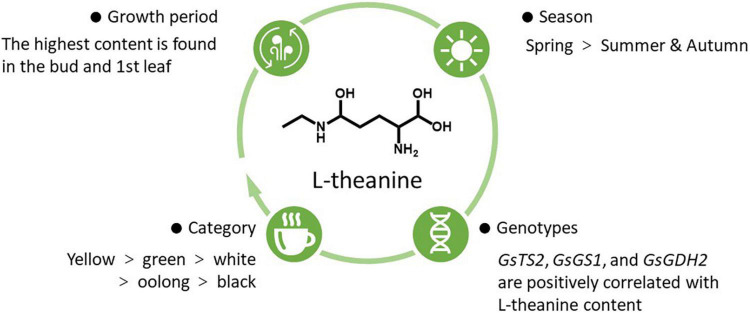
Influencing factors of L-theanine content in tea leaves.

L-theanine is widely distributed in different parts of the tea plants, and the content is different. L-theanine could be first produced in the roots of tea plants and then transported to the shoots (18). Its content in roots could be up to 6% of dry weight (19). Another study reported that tea leaves and roots had higher L-theanine contents than stems (20). Different influencing factors of L-theanine content in tea leaves are summarized in Figure 1.

Firstly, L-theanine content is variable among different tea categories. Through the quantitative analysis of 37 different varieties of tea, the average content of L-theanine in green, white, oolong, and black teas were 6.56, 6.26, 6.09, and 5.13 mg/g, respectively (21). Moreover, the content of L-theanine in albino yellow tea was higher than that in normal green tea, and the accumulation mechanism of albino yellow tea was associated with the slow catabolism of L-theanine (22). Secondly, L-theanine content was related to the expression of its metabolism-related genes. Among 17 identified genes related to L-theanine metabolism, the transcription levels of *GsTS2*, *GsGS1*, and *GsGDH2* were positively correlated with L-theanine content, while most other genes were negatively correlated (20). Thirdly, temperature and season also affect L-theanine content to a certain extent. It was found that melatonin could accelerate the photosynthesis of tea plants and increase the biosynthesis of L-theanine in tea leaves under sub-high temperature (35/30°C) (23). A combination of transcriptomics and metabolomics analysis showed that L-theanine content in spring was significantly higher than that in summer and autumn (24). Consistently, quantitative determination of 58 Chinese white tea showed that L-theanine content in the early spring-produced silver needle white tea was higher than that in the late spring-produced white peony white tea and autumn produced Shoumei white tea (25). Further analysis showed that the change of L-theanine content in different seasons was due to the effects of sunshine intensity on the photosynthesis of tea plants and then the expression of main transcription factors and structural genes (24). Finally, the content of L-theanine was affected by the growth period. Taking the leaves of tea at different stages (bud, 1st leaf, 2nd leaf, 3rd leaf, and old leaf) as the research object, it was found that the content of bud and 1st leaf was the highest and the content of L-theanine in leaves decreased gradually with the leave maturity (20). In addition, by comparing the fresh Jukro tea leaves at the growth stage of 40, 60, and 90 days, it was found that the content of L-theanine was the highest in the 60-day leaves (13).

To sum up, the content of L-theanine in tea leaves was higher. Even the content of L-theanine in the same tissue would vary due to several influencing factors. Among the six categories of tea, the content of L-theanine in albino yellow tea was the highest. Even in the same category of tea, L-theanine content also showed significant differences among genetic background, temperature and season, and growth periods.

## Health Benefits of L-Theanine

**FIGURE 2:**
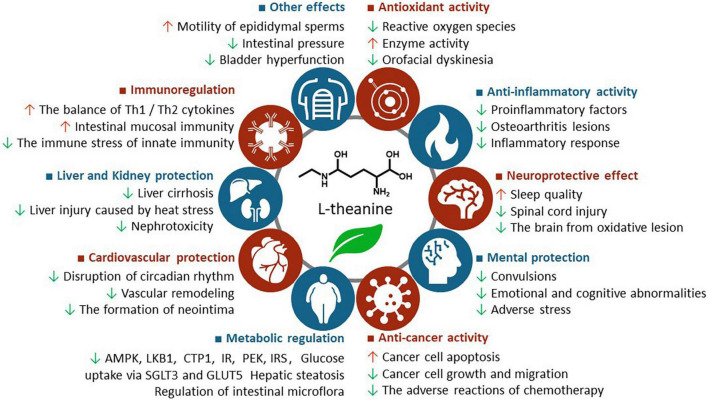
The health benefits of L-theanine. AMPK, adenosine 5′-monophosphate-activated protein kinase; CPT1, carnitine palmitoyltransferase 1; IR, insulin receptor; IRS, insulin receptor substrate; LKB1, liver kinase B1; PFK, phosphofructokinase.

L-theanine exhibits a variety of health benefits, such as antioxidant, anti-inflammatory, neuroprotective, anticancer, metabolic regulatory, cardiovascular protective, liver, and kidney protective, immune regulatory, as well as urogenital and intestinal protective effects, which are summarized in Supplementary Table 1 and briefly discussed below, with highlights about the potential mechanism of action (Figure 2).

### Antioxidant Activity

Recent studies reported that L-theanine exhibited good *in vitro* and *in vivo* antioxidant activities. In a neuronal-like rat pheochromocytoma cell model stimulated by cadmium oxide, L-theanine could reduce the synthesis of reactive oxygen species (ROS) and enhance the activity of antioxidant enzymes to weaken oxidative damage (26). It was reported that L-theanine showed antioxidant effects through adjusting the non-enzymatic activities, enhancing the activities and mRNA expression of catalase (CAT), and increasing superoxide dismutase (SOD) and glutathione peroxidase 1 (Gpx1) in enterotoxigenic *Escherichia coli* (ETEC)-infected mice (17). In a haloperidol (HAL)-induced rat model of orofacial dyskinesia (OD), the main feature of tardive dyskinesia (TD), it was found that L-theanine might have protective effects on OD due to its formidable antioxidant properties (27). In another study, L-theanine treatment reduced the levels of lipid peroxide and nitric oxide (NO) in OD rat models induced by HAL, thus improving the antioxidant capacity of the striatum (28). Combined with the existing evidence of animal studies, L-theanine may be able to treat human TD clinically through its antioxidant activity and regulating the activity of NO.

Generally, L-theanine has strong antioxidant activity, which can be associated with the regulation of the expression and activities of antioxidant-related enzymes.

### Anti-inflammatory Activity

The anti-inflammatory activity of L-theanine has been verified *in vitro* and *in vivo*. Through establishing an interleukin (IL)-1β-stimulated chondrocytes, it was found that L-theanine could inhibit the nuclear factor kappa B (NF-κB) pathway, thereby reducing the expression of proinflammatory factors, including cyclooxygenase-2 (COX-2), prostaglandin E2, inducible nitric oxide synthase, as well as NO, and protect the degradation of extracellular matrix (29). At the same time, L-theanine also significantly relieved osteoarthritis (OA) lesions in the anterior cruciate ligament transection-induced OA rat models (29). In addition, it was found that in 12-O-tetradecanoylphorbol-13-acetate (2.5 μg/ear)-induced ear edema mouse models, L-theanine could downregulate the expression of platelet endothelial adhesion molecule-1 (PECAM-1) and decrease the production of pro-inflammatory factors, including IL-1β, tumor necrosis factor-alpha (TNF-α), and COX-2, which were significantly expressed in neutrophils, thus improving the infiltration and activation of neutrophils (30). L-theanine was found to inhibit inflammation in rats with inflammatory bowel disease (IBD) induced by dextran sulfate sodium (DSS), and L-theanine treatment (200 mg/kg/day) could improve DSS-induced IBD through the molecular mechanisms related to cholesterol and retinol metabolism (31). In addition, the study on DSS-induced colitis in C57BL/6J male mouse models also confirmed that L-theanine could effectively inhibit intestinal inflammation (32). In rat models with intestinal stress induced by enterotoxigenic ETEC infection, the combined treatment of L-theanine with L-glutamine significantly decreased the expression of inflammatory factors, such as IL-1β, IL-6, and TNF-α (33). L-theanine was also found to reduce inflammation in lipopolysaccharide-induced mouse models, by normalizing the hyperactivity of the hypothalamus-pituitary-adrenal (HPA) axis and reducing the expression of inflammatory factors, including IL-1β, TNF-α, and IL-6, via inhibiting the NF-κB pathway (34). In ovalbumin-induced mouse asthma models, L-theanine treatment could reduce the transport of inflammatory cells to bronchoalveolar lavage fluid (BALF) and inhibit the infiltration of inflammatory cells via blocking the activation of NF-κB pathway and its downstream production of ROS, monocyte chemoattractant protein-1 (MCP-1), IL-4, IL-5, IL-13, TNF-α, and interferon (IFN)-γ in BALF (35).

In summary, the anti-inflammatory activity of L-theanine can be associated with inhibiting the expression of inflammatory factors and inflammation-related signaling pathways.

### Neuroprotective Effect

L-theanine has been reported with excellent neuroprotective effects on neuro injury. In a cell-based model induced by excessive dopamine, L-theanine exhibited neuroprotective effects on neuronal injury through the release of body fluid molecules from astrocytes, such as glutathione (36). Pretreatment of multipotential neural stem cells (NSCs) and C57BL/6J mice with L-theanine showed that L-theanine could alleviate the injury of NSCs induced by isoflurane and cognitive dysfunction of young mice, and the mechanism was related to the Akt/glycogen synthase kinase 3 beta (GSK3β) signaling pathway (37). In addition, since L-theanine was shown to have a relaxing effect and gamma-aminobutyric acid (GABA) was an important inhibitory neurotransmitter, the mixture of L-theanine and GABA had a positive synergistic effect on sleep behavior, including sleep quality and duration in caffeine-induced awake rats, and the mechanism might be that the mixture could promote the expression of GABA receptor, which was conducive to sleep (38). In another study, the mixture of L-theanine and Neumentix proprietary spearmint extract also regulated sleep disorders, prolonged sleep duration, significantly increased brain acetylcholine (Ach) and GABA concentrations, and decreased serotonin (5-HT) concentrations (39). Using the rat models of spinal cord injury (SCI), it was found that L-theanine could promote the recovery of behavioral motor function after SCI, and its potential neuroprotective mechanism may be related to the inhibition of posttraumatic oxidative reaction, neuroinflammation, and apoptosis (40). In rat models of orofacial dyskinesia induced by reserpine, L-theanine showed potential neuroprotective activities by reducing oxidative damage, neurotransmitter deficiency, neuroinflammation, and apoptosis (41). Moreover, by intraperitoneal injection of Aroclor 1254, brain oxidative stress and neurobehavioral changes were induced in rats (42). On this basis, the oral administration of L-theanine (200 mg/kg BW) could repair the normal brain structure, downregulate the expression of inflammatory cytokines, so as to protect the brain from the oxidative lesion (42). Further study on the brain injury model of mice induced by Cadmium (Cd) showed that L-theanine could protect mice from Cd-induced neurotoxicity, which was achieved by reducing the level of Cd in the brain and plasma, inhibiting the death of neurons in the cortex and hippocampus, improving the activities of SOD, GSH, and CAT in the brain, and most importantly, significantly alleviating the hyperphosphorylation of tau protein Ser199, Ser202, and Ser396 (26).

In addition, L-theanine could be cooperated with other substances to protect from neuro injury. A composite membrane was prepared by chemical grafting of L-theanine with graphene oxide, and it promoted the survival, proliferation, and neuronal differentiation of neural stem cells, suggesting that it might be used in the treatment of central nervous system injury (43). L-theanine and cystine, as supplements, performed well in the prevention of oxaliplatin-induced peripheral neuropathy in mouse models (44). This effect was further verified in human studies. Through the treatment of 28 patients with colorectal cancer, it was shown that daily oral intake of L-theanine and cystine could effectively reduce the damage of oxaliplatin-induced peripheral neuropathy. This was mainly because that oral intake of L-theanine and cystine could promote the synthesis of glutathione, which was a potential substance to prevent neuropathy (45). Furthermore, in a model of brachial plexus root avulsion created in Sprague Dawley (SD) rats, L-theanine combined with NEP1-40 observably accelerated nerve regeneration after brachial plexus root avulsion (46).

On the other hand, L-theanine has also been shown to be effective in treating neurodegenerative diseases. L-theanine could alleviate the memory impairment of the aging mouse by upregulating janus kinase 2 (JAK2)/activator of transcription 3 (STAT3), M1 muscarinic cholinergic receptor (mAChR), and extracellular signal-regulated kinase (ERK) signaling (47). L-theanine combined with luteolin could prevent symptoms similar to Alzheimer’s disease (AD) in rat models injected with amyloid-β (25–35) into the hippocampal CA1 region, and this was mainly associated with the improvement of hippocampal insulin signaling, norepinephrine metabolism, and the mitigation of neuroinflammation (48). L-theanine could also relieve the memory impairment and save the damage of hippocampal long-term potentiation in AD mice by activating the dopamine D1/5 receptor-protein kinase A pathway (49). Similarly, in the rat models of human Huntington’s disease (HD) induced by quinolinic acid, L-theanine alone could reduce the changes caused by QA (50). In rat HD models, neuropathological changes in the rat striatum were induced by 3-nitropropionic acid, and L-theanine exhibited neuroprotective effects, which mainly depended on not only inhibiting the production of harmful NO but also preventing the change of neurotransmitters in the striatum (51).

Therefore L-theanine has a good neuroprotective effect not only on improving cognitive and memory impairment, but also on preventing peripheral neuropathy and repairing nerves, and has a prominent influence on some neurodegenerative diseases, like AD and HD.

### Mental Protection

L-theanine also has positive effects on mental health. In mouse models, it was found that L-theanine could improve the anticonvulsive effect of pentobarbital sodium in a dose-dependent manner (52). In a mouse model of psychosocial stress, it was found that green tea had an anti-stress effect, which was due to the synergistic effect of L-theanine, epigallocatechin, and arginine, thus eliminating the antagonistic effect of caffeine and epigallocatechin gallate on psychological stress-induced adrenal hypertrophy (53). In adolescent male rat models exposed by the Delta-9-tetrahydrocannabinol (THC), L-theanine could strongly block the development of emotional and cognitive abnormalities associated with adolescent THC exposure, since L-theanine pretreatment could intercept THC-induced downregulation of local GSK-3 and Akt signaling pathway in the prefrontal cortex (PFC) (54). In addition, through behavioral tests and cerebrospinal fluid analysis in rats, L-theanine might change the levels of glutamate and methionine in the brain to improve the hippocampal activity, showing an antianxiety effect (55). Besides, the 30-day test score of 33 cats showed that L-theanine could alleviate all stress-related symptoms and eliminate the adverse stress performance after 15 days, and the effect was better after 30 days (56). In chronic unpredictable mild stress (CUMS) rat models, L-theanine could effectively improve the depressive-like behaviors of rats, which was regulated by monoamine neurotransmitters in the limbic-cortical-striatal-pallidal-thalamic-circuit related brain regions (57). L-theanine intake (6 mg/kg) could prevent brain atrophy and stress vulnerability in senescence-accelerated mice prone 10 (SAMP10) mice, with the mechanism of intaking L-theanine could block the expression changes of the transcription factor neuronal PAS domain protein 4 (*Npas4*) and Lipocalin 2 (*Lcn2*) in hippocampus and PFC of SAMP10 (58). Another study suggested that the administration of L-theanine ameliorated the depression-like behavior of stress-challenged SAMP10 mice (59).

Some clinical trials have also been carried out to verify the role of L-theanine in mental health. In terms of attention, a study involving 27 healthy adults showed that a high dose of L-theanine could improve the neurophysiological indexes of attention processing in a dose-dependent manner (60). Another study confirmed that L-theanine and caffeine had additive effects on cognition and attention in 20 healthy men (61). Furthermore, the test results of nine healthy adult males showed that L-theanine could reduce the allocation of neural resources to distractors, so attention would be more efficient in focusing on goals, and L-theanine and caffeine could cooperate to reduce mind wandering (62). In addition, in the study of Japanese men and women aged 50–69, L-theanine showed excellent performance in improving attention, and the working memory and executive function of the subjects were also enhanced (63). Furthermore, five boys (8–15 years old) with attention deficit hyperactivity disorder (ADHD) were treated with L-theanine and caffeine, and the result showed that L-theanine combined with caffeine could effectively treat ADHD-related injuries in sustained attention, inhibitory control, and overall cognitive performance (64). L-theanine has also shown good clinical effects in psychological and mental related aspects. For the purpose of assessing the influence of L-theanine on the mental and physical health of athletes, 20 college athletes were chosen for the study, and it was found that small amounts of L-theanine, caffeine, and tyrosine could boost the movement accuracy of athletes before and after exhaustive exercise (65). In another study, 30 subjects (9 men and 21 women, with the age of 48.3 ± 11.9 years old) without major mental illness were selected to evaluate the stress-related symptoms, sleep status, and cognitive function, and the result showed that L-theanine could facilitate the mental health of normal persons with stress-related diseases and cognitive disorder (66). On the other hand, 20 patients (4 males and 16 females, with the age of 41.0 ± 14.1 and 42.9 ± 12.0 years old, respectively) with major depressive disorders were selected as the research objects, and it was found that L-theanine (250 mg/day) treatment for 8 weeks was safe and effective to significantly mitigate the symptoms of depression, anxiety, somnipathy, and cognitive disorder (67). Besides, after taking L-theanine-containing beverage, the subjective stress response of 34 healthy adults aged 18–40 who were subjected to a multitasking cognitive stressor was significantly decreased, and the response of salivary cortisol to stressors was also decreased after positive treatment (68).

In summary, L-theanine shows excellent therapeutic effects on mental health, such as depression, stress, as well as emotional and cognitive function, and can also improve sleep condition and physical fitness to some extent.

### Anti-cancer Activity

Recent studies have demonstrated the anticancer activity of L-theanine in cell and animal models. Firstly, L-theanine could contribute to preventing the reproductive system cancers. In previous study, L-theanine and its derivatives, ethyl 6-bromocoumarin-3-carboxylyl L-theanine (TBrC), could effectively prevent the growth and migration of highly metastatic human cervical cancer cells, which was confirmed by *in vitro* and *in vivo* studies (69). They could decrease the expression and phosphorylation of epidermal growth factor receptor (EGFR), Met, Akt, and NF-κB in cervical cancer cells, and totally inhibit the EGFR/Met-Akt/NF-κB signaling pathway activated by hepatocyte growth factor (HGF) and epidermal growth factor (EGF) (69). Meanwhile, L-theanine and TBrC obviously inhibited the growth of cervical cancer in nude mice bearing tumors but showed no toxicity to mice. A recent study found that L-theanine had the therapeutic potential for metastatic prostate cancer (PCa), since L-theanine inhibited the epithelial-mesenchymal transition process of PCa by downregulating matrix metallopeptidase 9 (MMP9), N-cadherin, Vimentin, and Snail, and upregulating E-cadherin (70). Moreover, L-theanine also inhibited the transcription of MMP9 and Snail through weakening the ERK/NF-κB signaling pathway and p65 binding activity with MMP9 and Snail promoter region (70).

Secondly, L-theanine could induce or inhibit the digestive system cancers. It was shown that L-theanine (600 μg/mL) could induce apoptosis of tumor cells through the mitochondrial pathway in human HepG2 hepatoblastoma cells and HeLa adenocarcinoma cells (71). Furthermore, L-theanine and its semi-synthesized derivative (R)-2-(6,8-dibromo-2-oxo-2H-chromene-3-carboxamido)-5-(ethylamino)-5-oxopentanoic ethyl ester (DTBrC) also restrained the growth and migration of human hepatocellular carcinoma (HHC) cells in *in vitro*, *ex vivo*, and *in vivo* HHC models, and the mechanism of this effect was that L-theanine and DTBrC blocked the Met/EGFR/vascular endothelial growth factor receptor (VEGFR)-Akt/NF-κB pathways (72). Then, L-theanine alone or in combination with theobromine could effectively inhibit tumor production in male Wistar rats with colon cancer induced by dimethylhydrazine, and the mechanism of action was related to downregulating the Akt/mTOR (mammalian target of rapamycin) and JAK2/STAT3 pathways and increasing the mRNA and protein expression of tumor suppressor Smad2 (73).

In addition, L-theanine can also be used as an auxiliary measure to reduce some side effects of cancer treatment. L-theanine and cystine pretreatment (280 mg/kg for 5 days) could significantly enhance the weight loss and survival rate of rats after the irradiation, which may be connected with the inhibition of apoptosis and the enhancement of the proliferation of bone marrow cells (74). It was noteworthy that oral L-theanine could also weaken the adverse reactions of S-1 adjuvant chemotherapy (75).

In general, L-theanine could induce cancer cell apoptosis through the mitochondrial pathway, inhibiting the EGFR, NF-κB, and other signaling pathways, downregulating MMP9, or upregulating Smad2 in cancer treatment. In addition to acting directly on cancer cells, L-theanine also has beneficial effects in radiotherapy and chemotherapy.

### Metabolic Regulation

The absorption of nutrients is very important to human health, and L-theanine can effectively regulate metabolism. Pretreatment of RIN-m5F pancreatic beta-cell line with L-theanine increased the beta-cell mass and insulin production in a dose-dependent manner (76). In addition, L-theanine (50 μM) promoted the proliferation of human Sertoli cells (SCs) and increased its glucose metabolism (77). L-theanine can regulate metabolism in animal models. By observing serum insulin secretion and blood glucose concentration in rats, it was found that L-theanine downregulated the expression of *SGLT3* and *GLUT5* in the intestinal tract, leading to the inhibition of glucose uptake in the small intestine (78). In addition, L-theanine (100 mg/kg) could effectively regulate the metabolism of glucose, lipids, and proteins in SD rats, and the main mechanism was that L-theanine could upregulate the mRNA expression of phosphofructokinase (PFK), carnitine palmitoyltransferase 1 (CPT1), insulin receptor (IR), insulin receptor substrate (IRS), and liver kinase B1 (LKB1), and enhance the phosphorylation of adenosine 5′-monophosphate-activated protein kinase (AMPK) (79). The effects of L-theanine on metabolism were also supported by human studies. For example, serum ethylamine level was used as an indicator of L-theanine consumption, and the monitoring of 2,253 Japanese residents aged 40–79 without diabetes found that a higher level of serum ethylamine was significantly correlated with a lower risk of type 2 diabetes, suggesting a negative association between L-theanine and diabetes (80). L-theanine also played an effective role in diet-induced obesity. After oral administration of L-theanine, the metabolic activity of brown fat and subcutaneous white fat were increased, which significantly improved the obesity and hepatic steatosis of mice fed a high-fat diet (HFD), and the composition of intestinal microflora was also reasonably regulated (81).

These results suggest that L-theanine can regulate the metabolism of glucose, lipid, and protein by downregulating *SGLT3* and *GLUT5* expression and upregulating the mRNA expression of IR, PFK, IRS, especially since it has a positive health effect against diabetes and obesity.

### Cardiovascular Protection

L-theanine showed a positive effect on the cardiovascular system. It was reported that L-theanine could significantly inhibit the proliferation and migration of cultured vascular smooth muscle cells (VSMCs) induced by angiotensin II (82). The JAK2/STAT3 and ERK pathways were involved in the possible molecular mechanism. In addition, the pathogenesis of cardiovascular diseases (CVD) was also related to the dysregulation of circadian rhythm (82). In dexamethasone-induced rat VSMCs circadian gene expression models, L-theanine treatment showed that the expression of clock genes Bmal1, Cry1, Reverb alpha, and Per2 increased (83). At the same time, L-theanine could also upregulate a bunch of the rhythm genes and differential expression genes involved in vasoconstriction and actin cytoskeleton regulation pathways. Moreover, L-theanine could significantly inhibit the formation of neointima and prevent the transformation of VSMCs from contractile type to synthetic type in rat carotid artery balloon injury models (84). Further research showed that L-theanine had a potential preventive effect on neointimal hyperplasia and related vascular remodeling, mainly by inhibiting the phosphorylation of Elk-1 and activating mitogen-activated protein kinase-1 (MAPK-1) (84).

Collectively, L-theanine can block the JAK2, STAT3, and ERK1/2 pathways, regulate the expression of clock genes and rhythm genes and inhibit the formation of neointima, which makes L-theanine a potential cardiovascular beneficial substance.

### Liver and Kidney Protection

A number of studies have proved the positive effects of L-theanine on the liver. By adding L-theanine to the drinking water of cirrhotic rats established by carbon tetrachloride, it was found that L-theanine inhibited the expression of NF-κB, downregulated the pro-inflammatory cytokines (e.g., IL-1 and IL-6), and profibrotic mediators (e.g., transforming growth factor β and connective tissue growth factor), and promoted the expression of anti-inflammatory cytokine IL-10 and fibrinolytic enzyme metalloproteinases-13 (85). Therefore, L-theanine could effectively restrain liver cirrhosis in rats due to its anti-inflammatory and anti-fibrosis effects. In addition, L-theanine distinctly reduced the elevated serum aspartate aminotransferase (AST) and alanine aminotransferase (86) activities in ETEC infected mouse models (87). Further study showed that L-theanine could obviously increase the expression of Bcl-2 mRNA and protein, decrease the expression of Bax, a pro-apoptotic molecule, and decrease the phosphorylation of ERK1/2 and c-Jun NH2-terminal kinase (JNK1/2) MAPKs (87). In D-galactose-induced aging rats, L-theanine could protect the liver not only by reducing the levels of pro-inflammatory factors, such as IL-1β, TNF-α, and IL-6 but also by efficiently reducing the production of edema and vacuole induced by D-galactose (88). Liver injury is a side effect of heat stress. After intragastric administration of L-theanine before systemic heat exposure, heat-induced liver injury was also reduced in mice (89). In LPS-induced inflammatory mice, L-theanine treatment reduced the acute liver injury by inhibition of the concentrations of ALT and AST, the level of hepatic total superoxide dismutase (T-SOD), and malondialdehyde (MDA) (34). The molecular mechanism might be that L-theanine significantly reduced the release of IL-1β and TNF-α, and the phosphorylation of NF-κB, and increased the ratio of IL-10 to interferon (IFN)-γ.

With regard to kidney protection, in the doxorubicin (DOX) induced acute nephrotoxicity rat models, it was found that the treatment with L-theanine could attenuate the decrease of creatinine clearance, inhibit the production of lipid peroxidation in the kidney, and inhibit the reduction of glutathione content and SOD activity after DOX administration (90). Moreover, another study of DOX-induced nephrotoxicity in SD rats proved that L-theanine could protect the kidney by reducing the levels of oxidized glutathione (GSSG), gamma-glutamyltransferase 1 (GGT1), NF-κB p65, and the percentage of apoptosis indexes in the tissue and plasma, and increasing the levels of GSH and the activities of GPx, glutathione reductase (GR), and glutathione S-transferase (GST) (86). Furthermore, cecal ligation and perforation could lead to sepsis and damage the liver and kidney of SD rats, and L-theanine had a significant inhibitory effect on this kind of liver and kidney injury in a dose-dependent manner (91).

In general, the protective mechanism of L-theanine for the liver and kidney is to inhibit the NF-κB pathway, regulate pro-inflammatory cytokines, such as IL-1, IL-6, IL-10, and TNF, and finally regulate the activities of AST, ALT, T-SOD, and other related enzymes, to effectively protect the liver and kidney and deal with liver and kidney injury caused by different reasons.

### Immunoregulation

L-theanine has an excellent performance in immune regulation. In the SD rat models, daily intragastric administration of L-theanine solution (400 mg/kg) could increase the weight of the spleen, modify the balance of Th1/Th2 cytokines, reduce the level of serum corticosterone, increase the level of dopamine and 5-HT in the brain, and regulate the mRNA expression of phospholipase C isomers in the heart, finally improving the immune function (92). Moreover, in another study, L-theanine effectively regulated the secretion of cytokines such as IFN-γ, IL-2, IL-4, IL-10, IL-12, and TNF-α except for IL-6 by activating the mRNA and protein expression of Ras-related protein Rap-1A (Rap1A), 3-hydroxy-3-methylglutaryl-CoA reductase (HMGCR), and farnesyl diphosphate synthase (FDPs) in the mevalonate synthesis pathway of rat splenic lymphocytes (93). In addition, L-theanine treatment decreased the mRNA expression of Toll-like receptors (e.g., TLR-2 and TLR-4) and cytokines (e.g., IFN-α, IFN-γ, and IL-2) in broilers (94). Furthermore, a 28-day feeding study of SD rats showed that L-theanine could increase the content of total short-chain fatty acids and regulate intestinal mucosal immunity based on dietary fiber feeding (95). Whereafter, L-theanine could regulate the innate immunity of mice with immune stress induced by ETEC, mainly by inhibiting the overexpression of nucleotide-binding oligomerization domain, IL-1β, and TNF-α, partially reducing the protein level of NF-κB p65, and suppressing the phosphorylation of ERK1/2 (96). In another study, it was found that L-theanine antagonized cannabinoid receptor 1 and inhibited its activity, relieved the inhibition of cannabinoid receptor 1 on COX-2 expression, reduced the pro-inflammatory factor TNF-α, and enhanced the anti-inflammatory factor IL-10, making L-theanine show a significant regulatory effect on the immune function of normal and E44813-stressed rats (97). Additionally, in the study of Polish rowing team members, it was found that L-theanine supplementation could contribute to the reduction of IL-10 concentration after exercise, which had an advantageous influence on the destruction of Th1/Th2 balance of top athletes (98).

In a word, L-theanine can regulate the balance of Th2/Th1 cytokines and the content of related substances. The main mechanism is closely related to the expression of protein and mRNA of action factors.

### Urogenital Protection

L-theanine also exhibits protection in the urogenital system *in vitro* and *in vivo*. In urethane-anesthetized female Wistar rats, L-theanine could reduce substance P-induced bladder hyperfunction through inhibiting pro-inflammatory protein kinase C (PKC)/ERK/NF-κB/intercellular adhesion molecule 1 (ICAM-1)/IL-33 signaling, oxidative stress, apoptosis, autophagy (99). It was noted that when L-theanine, caffeine, and EGCG were supplemented in the culture medium, the motility of rat epididymal sperms was increased after 72 h of incubation at room temperature (100). More than that, studies on SCs found that L-theanine (50 μM) could promote the proliferation and glucose metabolism of human SCs to maintain the Krebs cycle, which was very important to prevent spermatogenesis disruption (77). On the whole, L-theanine can block bladder hyperfunction and protect spermatogenesis.

### Intestinal Protection

Recent studies suggest that L-theanine exhibits intestinal protective effects in animal models. When L-theanine was added to the diet of broilers, L-theanine had a beneficial effect on the intestinal microbiota, with increases in beneficial microorganisms, such as Lactobacillus, while inhibiting harmful microorganisms, such as Clostridium (94). Another study also validated that L-theanine could improve the intestinal development and health status of broilers, and the relative weight of duodenum, jejunum, and ileum increased, and the villus height and glutathione peroxidase activity of jejunum showed a linear or quadratic increase, and also enhanced the mRNA levels of intestinal amino acid and peptide transporters (101). It was also found that L-theanine could alleviate the intestinal pressure and stabilize the healthy intestinal tract in the stressed rat models established by E44813 infection, mainly by significantly enhancing the synthesis of glutamine and increasing the villus height and crypt depth of the intestinal tract (33). Overall, the intestinal protection of L-theanine can mainly be associated with the regulation of intestinal microbiota and the reduction of enterotoxin-mediated intestinal damage.

## Food Applications of L-Theanine

**FIGURE 3:**
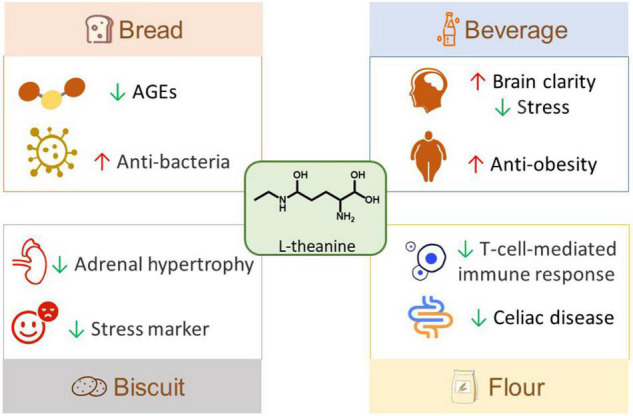
Food applications of L-theanine. AGEs, advanced glycation end products.

L-theanine has some applications in foods and this kind of functional food has a positive effect on health and is very popular with consumers (Figure 3). L-theanine powder was obtained from decaffeinated tea and then made into theanine bread and other baked foods (102). Tea powder was used to prepare L-theanine enriched fractions (TEF), which could prevent the formation of fluorescent advanced glycation end-products, therefore, TEF was used to make healthy and delicious L-theanine bread (103). In addition, the concentrated L-theanine was added to wheat bread, which could inhibit E. coli and extend the shelf-life of bread for 1 day (40). Besides, due to the anti-stress effect of L-theanine, a nutritional beverage was made based on L-theanine, and the test results showed that the beverage could significantly reduce the subjective stress of 36 participants responding to multi-task cognitive stressors (68). Moreover, the cold-water brewed green tea, a new type of functional low-calorie beverage, was made at 30°C and contained a large number of L-theanine, catechin, gallic acid, and other bioactive ingredients, and it was found to significantly alleviate obesity and regulate the intestinal flora of HFD mice (104). A nootropic beverage containing L-theanine, pine bark, and blackcurrant could reduce mental fatigue in the sports environment, maintain brain clarity, improve the total score and accuracy, and effectively control the tension of athletes (105). In order to alleviate pressure, L-theanine was added into mango sorbet to make functional food, and the study proposed that the influence of food matrix should be considered when establishing functional food ingredients (106). Moreover, eating Matcha biscuits containing L-theanine also verified the pressure-reducing effect of L-theanine through animal experiments and clinical trials (107). Furthermore, a wheat flour rich in L-theanine was developed, and it could forcefully weaken the immune response mediated by T cells stimulated by gluten in the intestine of patients with celiac disease, and retain the function of gluten (108). This suggests its potential application in gluten-containing food products.

At present, the food application of L-theanine is mainly in beverages and bread. L-theanine has attracted much attention due to its various biological activities, suggesting its potential for formulating functional food products. In the future, more applications of L-theanine in foods should be explored, and its dosage and processing in products should be further studied.

## Conclusion and Perspectives

L-theanine is a special free amino acid in tea, which is widely distributed in tea plants. The content of L-theanine in different kinds of tea is varying, with the climate and growth period affecting its content. As one of the main components of tea, L-theanine also has a variety of health benefits and some applications in foods as mentioned above. In the future, the following points are worthy of attention. Firstly, the mechanism of L-theanine on tea flavor should be further studied, and the changing trend of L-theanine content in different fermentation stages should be explained. Secondly, although a number of studies have confirmed the health benefits of L-theanine *in vitro* and *in vivo*, human-based research is still limited, and more clinical trials should be guaranteed to evaluate the health benefits of L-theanine. Overall, L-theanine exhibits plenty of beneficial functions and can be a promising functional additive or supplement in the food and nutritional industry.

## Author Contributions

R-YG and D-TW: conceptualization, supervision, and funding acquisition. M-YL and H-YL: writing—original draft preparation. D-TW, AK, H-BL, AG, FG, HL, and R-YG: writing—review and editing. R-YG: project administration. All authors read and agreed to the published version of the manuscript.

## Conflict of Interest

The authors declare that the research was conducted in the absence of any commercial or financial relationships that could be construed as a potential conflict of interest.

## Publisher’s Note

All claims expressed in this article are solely those of the authors and do not necessarily represent those of their affiliated organizations, or those of the publisher, the editors and the reviewers. Any product that may be evaluated in this article, or claim that may be made by its manufacturer, is not guaranteed or endorsed by the publisher.
