# Supplementary material for: L-Theanine: A Unique Functional Amino Acid in Tea (Camellia sinensis L.) With Multiple Health Benefits and Food Applications
Source: Front Nutr. 2022 Apr 4;9:853846. doi: 10.3389/fnut.2022.853846 (PMC9014247; doi:10.3389/fnut.2022.853846)
Supplement: Supplementary file 1 [file Table_1.docx]

**Table 1** Health benefits and related mechanisms of L-theanine.

| **Study type** | **Dosages** | **Experimental models** | **Main effects** | **Mechanisms of action** | **References** |
| --- | --- | --- | --- | --- | --- |
|  |  |  | Antioxidant activity |  |  |
| *In vitro* | 100 and 200 mg/kg/day for 8 weeks | Neuronal-like rat pheochromocytoma cell models | Exerting antioxidant activity | SOD↑  The activity of antioxidant enzymes, ROS↓ | (26) |
| *In vivo* | 100, 300, and 500 mg/kg | BALB/c female mouse models of oxidative stress infected by enterotoxigenic ETEC | Exerting antioxidant activity | SOD, Gpx1↑ CAT↓ | (17) |
| *In vivo* | 100 and 300 mg/kg | HAL-induced OD rat models | Exerting antioxidant activity | LPO ↓ | (27) |
| *In vivo* | 10, 30, and 50 mg/kg | HAL-induced OD rat models | Exerting antioxidant activity | LPO, NO↓ | (28) |
|  |  |  | Anti-inflammatory activity |  |  |
| *In vitro* | 50, 100, and 200 mg/kg | IL-1β-stimulated primary chondrocytes from rats | Inhibiting inflammatory response and reducing inflammatory cytokines | COX-2, PGE-2, MMP-3, MMP-13, iNOS and NO↓ | (29) |
| *In vivo* |  | ACLT-induced osteoarthritis rat models |  | COX-2, PGE-2, C2C, CTX-II, iNOS, NO and NF-κB pathway↓ |  |
| *In vivo* | 5, 50, and 250 mM | 12-O-tetradecanoylphorbol-13-acetate (TPA, 2.5 μg/ear)-induced ear edema female CD1 mouse models | Relieving acute cutaneous inflammation | PECAM-1, IL-1β, TNF-α and COX-2↓ | (30) |
| *In vivo* | 0, 50, 200, and 800 mg/kg/day | DSS-induced IBD SD male rat models | Exerting anti-inflammatory activity | Regulating cholesterol and retinol metabolism | (31) |
| *In vivo* | 0.05% and 0.1%(w/v) | DSS-induced colitis C57BL/6J male mouse models | Reducing intestinal inflammation | iNOS, COX-2, TLR-2/-4, /-6, /-9, IL-1β/-6, TNF-α, LPS↓ | (32) |
| *In vivo* | 300, 600, and 900 mg/kg | Stressed male SD rat models infected with ETEC | Reducing inflammation | Regulating the synthesis of glutamine | (33) |
| *In vivo* | 20 and 40 mg/kg/day | LPS-induced inflammation male ICR mouse models | Reducing liver inflammation | NF-κB pathway, IL-1β/-6, TNF-α↓ IL-10/IFN-γ ration↑ Normalization of HPA axis hyperactivity | (34) |
| *In vivo* | 10, 50, and 100 mg/kg/day | Ovalbumin-induced asthma female ICR mouse models | Alleviating airway inflammation | MCP-1, immunoglobulin E, IL-4/-5/-13, TNF-α, IFN-γ, ROS, NF-κB pathway↓ | (35) |
|  |  |  | Neuroprotective effect |  |  |
| *In vitro* | 5, 50, and 500 μM | Primary astrocytes and neuro cells | Repairing neuronal damage | GSH↑ | (36) |
| *In vivo* | 4 mg/kg/day for 14 days | Male ICR mice | Exerting neuroprotective effect | Striatal GSH ↑ |  |
| *In vitro* | 10 and 100 μM | Multipotential neural stem cells | Alleviating cells damage | Caspase-3↑ p-Akt, p-GSK-3β↓ | (37) |
| *In vivo* | 10 mg/kg | C57BL/6J mice | Alleviating cognitive deficits | Akt/GSK-3β pathway |  |
| *In vivo* | 20, 30, and 40 mg/kg | Pentobarbital-induced sleep male ICR mouse models and SD rat models | Promoting sleep | Changing GABA and/or glutamate receptor expression | (38) |
| *In vivo* | 27, 82, and 245 mg/kg | Pentobarbital-/barbital-induced sleep male BALB/C mouse models | Regulating sleep disorders | Ach, GABA↑ 5-HT↓ | (39) |
| *In vivo* | 10 and 30 mg/kg | Compression injury male Wistar rat models | Restoring behavioral motor functions | NO, MDA↓ | (40) |
| *In vivo* | 100 and 300 mg/kg for 14 days | Reserpine -induced behavioral and neurochemical dysfunction in male Wistar rat models | Exerting neuroprotective effect | LPO↓ Catecholamines↑ | (41) |
| *In vivo* | 200 mg/kg for 30 days | Aroclor 1254-induced oxidative stress in male Albino rat models | Improving the neurobehavioral activities and memory function | Acetylcholinesterase, TNF-α, IL-6↓ CAT, SOD, GR, GPx, GSH↑ | (42) |
| *In vivo* | 100 and 200 mg/kg/day for 8 weeks | Cadmium-induced brain injury Male ICR mouse models | Avoiding neurotoxic damage | GSK-3β, MDA, Akt/mTOR↓ GSH, CAT, GSH-peroxidase↑ | (26) |
| *In vivo* | 80 mg/kg/day for 4 weeks | Oxaliplatin-induced peripheral neuropathy male SD rat models | Ameliorating chronic neuropathic symptoms | Protective effects against axon disorders (axonopathy) | (44) |
| Clinical trial | 280 mg/day | 28 colorectal cancer patients | Attenuating oxaliplatin-induced peripheral neuropathy | / | (45) |
| *In vivo* | 100 mg/kg/day for 2 weeks | Female SD rat models of brachial plexus root avulsion | Promoting nerve regeneration | Antagonism of myelin inhibition | (46) |
| *In vivo* | 4 mg/kg/day | *Klotho* mutant mouse models | Improving memory dysfunction | p-JAK2/p-STAT3, M1 mAChR, ERK 1/2 signaling↑ | (47) |
| *In vivo* | 50 and 100 mg/kg | Alzheimer’s disease male SD rat models injected with amyloid-β (25-35) in hippocampal CA1 region | Ameliorating memory impairment | Hippocampal insulin signaling↑ TNF-α, norepinephrine↓ | (48) |
| *In vivo* | 0.1 and 0.4 mg/mL | Alzheimer’s disease APP/PS1 male mouse models | Ameliorating memory impairment and hippocampal long-term potentiation | Dopamine 1/5 receptor-PKA pathway ↑ | (49) |
| *In vivo* | 25 and 50 mg/kg/day for 21 days | QA-induced male Wistar rat models | Exerting neuroprotective effect | TNF-α, IL-6/-1β↓ dopamine, 5-HT↑ | (50) |
| *In vivo* | 25 and 50 mg/kg/day for 21 days | 3-nitropropionic acid -induced Huntington’s disease -like symptoms male Wistar rat models | Inhibiting neurotoxicity | TNF-α, IL-6/-1β↓ dopamine, 5-HT↑ | (51) |
|  |  |  | Mental protection |  |  |
| *In vivo* | 0.5, 1.0, and 2.0 g/kg | ICR mouse models | Exerting anticonvulsion effect | AMPA receptor, glycine↓ | (52) |
| *In vivo* | 0.32 and 3.2 mg/kg | Male ddY mouse models | Exerting anticonvulsion effect | / | (53) |
| *In vivo* | 10 mg/kg | THC-induced male SD rat models | Mitigating the neuropsychiatric side-effects | GSK-3 and Akt signaling pathways↑ | (54) |
| *In vivo* | 0.4 mg/kg | Anxiety and depressive disorders male Wistar rat models | Exerting anxiolytic effect | Glutamate↓ Methionine↑ | (55) |
| *In vivo* | 50 mg/day for 30 days | 33 Cats | Alleviating stress-related symptoms | / | (56) |
| *In vivo* | 2 mg/kg/day for 21 days | Chronic unpredictable mild stress male SD rat models | Exerting antidepressant effect | 5-HT, dopamine↑ (LCSPT‐circuit related brain regions) | (57) |
| *In vivo* | 6 mg/kg | Stress-loaded male SAMP10 and ddY mouse models | Preventing stress-induced brain atrophy | Modifying early stress responses (*Npas4*, *Lcn2*) | (58) |
| *In vivo* | 6 mg/kg | Stress-loaded male SAMP10 and ddY mouse models | Exerting antidepressant effect | KYN↑ Carnosine↓ | (59) |
| Clinical  trials | 100, 200, and 400 mg | 27 young adults | Enhancing neurophysiological indices of attentional processing | / | (60) |
| Clinical  trials | 200 mg | 20 male adults | Enhancing attention | / | (61) |
| Clinical  trials | 200 mg | 9 male adults | Enhancing attention | / | (62) |
| Clinical  trials | 100.6 mg for 12 weeks | 29 adults | Enhancing attention | / | (63) |
| Clinical  trials | 2.5 mg/kg | 5 boys with ADHD | Improving in sustained attention and overall cognition composite | / | (64) |
| Clinical  trials |  | 20 male athletes | Improving movement accuracy | / | (65) |
| Clinical  trials | 200 mg/day for 4 weeks | 30 adults | Promoting mental health | / | (66) |
| Clinical  trials | 250 mg/day for 8 weeks | 20 adults | Promoting neuronal specification | *Slc38a1*↑ | (67) |
| Clinical  trials | 200 mg | 34 adults | Exerting anti-stress effect | / | (68) |
|  |  |  | Anti-cancer activity |  |  |
| *In vitro* | 600 μg/mL | Human hepatoblastoma HepG2 cells, human cervical carcinoma HeLa cells, human hepatic L02 cells, human embryonic kidney 293 cells and rat cardiac myoblast H9c2 cells | Inducing tumor cells death | Mitochondrial pathway Caspase-3/-9↑ | (71) |
| *In vitro* | 16−250 μM for 29 days | Human cervical cancer cell lines HeLa and CaSki | Inhibiting tumor cells growth and migration | EGFR, Met, Akt, NF-κB↓ | (69) |
| *In vivo* | 60−160 mg/kg for 29 days | BALB/c mouse models | Treating cervical cancer |  |  |
| *In vitro* | 87 mg | The cell lines of human hepatocellular carcinoma SMMC7721 and HepG2 | Inhibiting human hepatocellular carcinoma growth and migration | Met, EGFR, VEGFR2↓ | (72) |
| *In vivo* | 80 mg/mL/kg | Tumor female C57/BL6 mouse models | Inhibiting tumor growth |  |  |
| *In vitro* | 0−300 μM | Human prostate cancer cell lines PC3, DU145, LNCaP, 22RV1, WPMY-1 and MCF-10A | Inhibiting prostate cancer cells invasion and migration | MMP9, N-cadherin, Vimentin, Snail, ERK/NF-κB pathways↓ E-cadherin↑ | (70) |
| *In vivo* | 80 mg/kg for 10weeks | Male BALB/c mouse models Male NOD-SCID mouse models | Inhibiting prostate cancer cells invasion and migration |  |  |
| *In vivo* | 400 mg/kg | Dimethylhydrazine - induced colon cancer male Wistar rat models | Preventing colon carcinogenesis | JAK2/STAT3, Akt/mTOR↓ Smad mRNA↑ | (73) |
| *In vivo* | 280 mg/kg for 5 days | Male Wistar rat models | Attenuating acute injury after irradiation | Apoptosis↓ Cell proliferation↑ | (74) |
| Clinical  trials | 280 mg for 5 weeks | Patients who underwent R0 surgery for either colon or gastric cancer at the surgery department | Attenuating the adverse events of S-1 adjuvant chemotherapy | / | (75) |
|  |  |  | Metabolic regulation |  |  |
| *In vitro* | 37.5, 75, 100, and 300 μm | RIN m5F cell models | Preventing or treating diabetes | Protecting pancreatic β-cells Insulin↑ | (76) |
| *In vitro* | 5 and 50 μM | Human Sertoli cell models | Promoting glucose metabolization | ATP, Cellular components synthesis↑ | (77) |
| *In vivo* | 50, 200, and 400 mg/kg /day for 2 weeks | SD rat models | Hypoglycemic effect | *SGLT3* and *GLUT5* mRNA↓ Intestinal transporters↑ | (78) |
| *In vivo* | 100, 200, and 400 mg/kg for 28 days | Male SD rat models | Regulating glucose, lipid, and protein metabolism | Insulin and AMPK and their downstream signaling pathways | (79) |
| Clinical  trials | 8 mg | 2,253 community-dwelling Japanese individuals without diabetes were monitored for 7 years | Preventing type 2 diabetes | Serum ethylamine↑ | (80) |
| *In vivo* | 30 mg/kg/day for 12 weeks | C57BL/6 male HFD mouse models | Improving obesity | Fat browning, brown adipose tissue, white adipose tissue↑ | (81) |
|  |  |  | Cardiovascular protection |  |  |
| *In vitro* | 5, 50, and 100 μM | Angiotensin II-induced VSMCs | Protecting cardiovascular | JAK/STAT3 and ERK pathways↑ | (82) |
| *In vitro* | 40 μM | Dexamethasone-induced circadian gene expression models in rat VSMCs | Modulating vascular circadian system | *RhoA, Rock2, CPI-17*↓ | (83) |
| *In vitro* | 100, 200, and 400 μM | VSMCs | Regulating vascular homeostasis | VSMCs phenotypic transformation↓ | (84) |
| *In vivo* | 50 mg/kg/day | Carotid-artery balloon-injury SD rat models | Treating vascular diseases with injury | Neointimal formation, Elk-1↓ MAPK 1↑ |  |
|  |  |  | Liver and kidney protection |  |  |
| *In vivo* | 8 mg/kg for 8 weeks | Carbon tetrachloride-induced hepatic cirrhosis male Wistar rat models | Exerting antifibrotic effect | NF-κB, IL-1/-6, TGF-β, CTGF↓ IL-10, fibrolytic enzyme metalloproteinase-13↑ | (85) |
| *In vivo* | 100, 300, and 500 mg/kg for 30 days | ETEC-infected mouse models | Attenuating liver injury | AST, ALT, ERK1/2 and JNK1/2 MAPK phosphorylation signaling pathways↓ Bcl-2↑ | (87) |
| *In vivo* | 100, 200, and 400 mg/kg/day for 8weeks | D-galactose-induced male SD rat models | Ameliorating liver aging | IL-1β/-6, TNF-α, NF-κB↓ IL-4/-10, FOXO1 mRNA↑ | (88) |
| *In vivo* | 100 and 200 mg/kg | Male ICR mouse models | Attenuating liver injury | Hepetic heat shock proteins↓ Normalizing HPA axis hyperactivity | (89) |
| *In vivo* | 20 and 40 mg/kg/day | LPS-induced inflammation male ICR mouse models | Reducing liver inflammation | NF-κB pathway, IL-1β/-6, TNF-α↓ IL-10/IFN-γ ration↑ Normalization of HPA axis hyperactivity | (34) |
| *In vivo* | 10 mg/kg | Doxorubicin-induced acute nephrotoxicity male SD rat models | Inhibiting acute renal toxicity | Suppressing oxidative stress | (90) |
| *In vivo* | 200 mg/kg/day for 5 days | Doxorubicin-induced nephrotoxicity male SD rat models | Inhibiting nephrotoxicity | GSSG, GGT1, NF-κB p65↓ GSH, GPx, GR, GST↑ | (86) |
| *In vivo* | 250 and 750 mg/kg | Cecal ligation and puncture-induced sepsis male SD rat models | Inhibiting liver and kidney injury | iNOS, TNF/IL-10 ratio, Caspase-3↓ | (91) |
|  |  |  | Immunoregulation |  |  |
| *In vivo* | 50, 200, and 400 mg/kg | SD rat models | Improving immune function | ILs-4/-6/-10, IL-4/INF-γ ratio↓ Dopamine, 5-HT↑ | (92) |
| *In vivo* | 0.25, 1, and 4 Mm | Male SD rat models | Exerting immunomodulatory effects | Mevalonate biosynthetic pathway Rap1A, HMGCR↑ | (93) |
| *In vivo* | 100, 200, and 300 mg/kg | Broiler chicks | Exerting immunomodulatory effects | TLR-2/-4, TNF-α, IFN-γ, IL-2↓ | (94) |
| *In vivo* | 100, 300, and 500 mg/kg | Male SD rat models | Affecting intestinal mucosal immunity | Regulating short-chain fatty acid metabolism | (95) |
| *In vivo* | 100, 300, and 500 mg/kg | ETEC-induced immune stressed female BALB/c mouse models | Regulating innate immunity | NOD1/2-NF-κB and NOD1/2-MAPK pathways↓ | (96) |
| *In vivo* | 600 mg/kg | ETEC-induced stressed male SD rat models | Regulating immune function | TNF-α↓ IL-10↑ | (97) |
| Clinical  trials | 150 mg | 20 rowers | Modulating immune system | IL-2/IL-10 and IFN-γ/IL-10 ratios↑ | (98) |
|  |  |  | Urogenital protection |  |  |
| *In vivo* | 5 and 15 mg/kg for 2 weeks | Urethane-anesthetized female Wistar rat models | Ameliorating bladder hyperactivity | PCK.ERK/NF-κB/ICAM-1/IL-33 signaling↓ | (99) |
| *In vivo* | 19 μg/mL | Male Wistar rat models | Improving spermatozoa preservation | Protein oxidation | (100) |
| *In vivo* | 5 and 50 μM | Human Sertoli cell models | Preventing spermatogenesis disruption | Glycolysis, glutaminolysis | (77) |
|  |  |  | Intestinal protection |  |  |
| *In vivo* | 100, 200, and 300 mg/kg | Broiler chicks | Regulating intestinal bacteria | *Lactobacillus*↑ *Clostridium*↓ | (94) |
| *In vivo* | 300, 600, 900, and 1500 mg/kg | Male Ross 308 broilers | Improving intestinal development | Peptides, amino acids↑ | (101) |
| *In vivo* | 300, 600, and 900 mg/kg | Stressed male SD rat models infected with ETEC | Stabilizing intestinal tract | Increasing intestinal villi height and crypt depth | (33) |

Abbreviations: SOD, superoxide dismutase; ROS, reactive oxygen species; ETEC, Escherichia coli; Gpx1, glutathione peroxidase 1; CAT, catalase; HAL, haloperidol; OD, orofacial dyskinesia; LPO, lipid peroxide; NO, nitric oxide; IL, interleukin; COX-2, cyclooxygenase-2; PGE-2, prostaglandin E2; MMP, matrix metallopeptidase; iNOS, inducible nitric oxide synthase; ACLT, anterior cruciate ligament transection; C2C, Col2-3/4C-terminalcleavageproductoftype II collagen; CTX- II, crosslinked C-telopeptides of Type II collagen; NF-κB, nuclear factor kappa B; PECAM-1, platelet endothelial adhesion molecule-1; TNF-α, tumor necrosis factor-alpha; DSS, dextran sulfate sodium; IBD, inflammatory bowel disease; SD, Sprague Dawley; TLR, toll like receptor; LPS, lipopolysaccharide; GSH, glutathione; IFN-γ, interferon-γ; HPA, hypothalamus-pituitary-adrenal; MCP-1, monocyte chemoattractant protein-1; Akt, protein kinase B; GSK-3β, p-glycogen synthase kinase-3 beta; GABA, gamma-aminobutyric acid; Ach, acetylcholine; 5-HT, serotonin; MDA, malondialdehyde; LPO, lipid peroxidation; GR, glutathione reductase; GPx, glutathione peroxidase; mTOR, mammalian target of rapamycin; JAK2, janus kinase 2; STAT3, transcription 3; M1 mAChR, M1 muscarinic cholinergic receptor; ERK, extracellular signal-regulated kinase; PKA, protein kinase A; QA, quinolinic acid; AMPA, alpha-amino-3-hydroxy-5-methyl-4-isoxazolepropionic acid; THC, Delta-9-tetrahydrocannabinol; LCSPT, limbic–cortical–striatal–pallidal–thalamic; SAMP10, senescence-accelerated mice prone 10; Npas4, neuronal PAS domain protein 4; Lcn2, Lipocalin 2; KYN, kynurenine; ADHD, attention deficit hyperactivity disorder; EGFR, epidermal growth factor receptor; VEGFR, vascular endothelial growth factor receptor; AMPK, adenosine 5’-monophosphate -activated protein kinase; HFD, high-fat diet; VSMCs, vascular smooth muscle cells; MAPK, mitogen-activated protein kinase; TGF-β, transforming growth factor β; CTGF, connective tissue growth factor; AST, aspartate aminotransferase; ALT, alanine aminotransferase; GSSG, glutathione; GGT1, gamma-glutamyltransferase 1; GST, glutathione S-transferase; NOD, nucleotide-binding oligomerization domain; PCK, protein kinase C; ICAM-1, inter-cellular adhesion molecule 1.
